# The Hospital

**Published:** 1918-03-02

**Authors:** 


### INSTITUTIONAL WORKER

Being a Special Supplement to "The Hospital"

### OUR BUREAU OF INFORMATION.

### Rules for Correspondents.

1. Every letter must be accompanied by the coupon to be cut from the back cover (inside page) of The Hospital, current issue, and must contain the name and address of the correspondent with pseudonym for publication if desired. Replies by post cannot be given save under exceptional circumstances at the Editor's discretion.

2. Letters from Approved Homes in reply to special needs published in the Bureau should state terms and full particulars, and be sent prepaid under cover to the Editor of the Bureau with name written across coupon for identification.

3. Proprietors of Homes which have not yet been entered on the List of Approved Homes, but have spare accommodation likely to suit special needs, are invited to write for an application form for registration. The fee for registration, which includes two announcements of the Home in the Bureau and other privileges, is 10s.

4. All communications to be addressed to the Editor of The Hospital, 28 Southampton Street, Strand, London, W.C. 2, and marked "Bureau of Information."

### INSTITUTION FACTS AND FIGURES.

### Extending a Hospital Engineer's Work.

### Answer to January Question.

By F. Squires.

The question was:

**In what ways can a hospital engineer be useful in addition to his routine work of attending to the heating apparatus?**

The engineering staff of a general hospital (200-300 beds) should consist of the engineer himself and one or more stokers, according to the centralisation or otherwise of the heating plant. The engineer's primary duty should be to supervise the heating apparatus. He should have a fair working knowledge of electricity, and be capable of undertaking general repairs.

Extra Work to be Undertaken.

The engineer should accompany the house governor on his periodical inspections of the structure, and should be able to carry out temporary repairs to roofing and other structural items. He should be responsible for the proper condition of all fire-extinguishing apparatus and the regular re-charging of the chemical extinguishers. He should keep clear all pipes, drains, and sewers, and supervise the disinfection of all drains on the premises and in the grounds. The recording of electricity, gas, and water consumption should be part of his duty. He should replace all damaged electric lamps, and keep records of the "lives" of lamps in the different departments; the various repairs of switches, bell-pushes, and the re-charging of cells in connection therewith should be undertaken by him. The engineer should promptly attend to washers of taps, especially those of the hot-water supply, to obviate unnecessary waste of steam. Many metal articles of domestic ware can be repaired on the premises with economical results.

Simple surgical appliances, such as metal splints of simple design, should be made, altered, and repaired by him when necessary. It might here be mentioned that it is, in the writer's opinion, inadvisable to allow the engineer to attempt the repair of surgical instruments or the more scientific parts of the *x*-ray installation, as it is distinctly risky to place an instrument in the surgeon's hand that has been repaired by unskilled workmanship. The engineer's practical experience should be taken advantage of when the alteration or expansion of the buildings is under consideration.

At the institution the writer has in mind the engineer undertakes all the items above enumerated, without detriment to his duties in connection with the heating apparatus.

### Crockery Washing in a Nurses' Home.

### Answer to January Question.

By "A. M."

The question was:

**Describe the cleanest, quickest, and most careful method of washing and drying crockery in a nurses' home to accommodate 180 persons.**

The first essentials for clean and quick washing-up of crockery are a large sink, a plentiful supply of hot water, soap, soda or soap-powder, a dish-cloth, a mop on a stick, and six or eight drying-cloths. The washing-up should be done in a large pulp bowl placed inside the sink, as more crockery is broken by being carelessly knocked together in or against the sides of a sink than in any other way. The taps are more convenient if fitted at the end (right-hand side) than in the centre. A pulp bowl is better than enamel, being softer.

Assuming that there is one washer-up, who also washes cutlery and silver, the following method is good. Place knives in jugs containing hot soapy water (water not to reach handles), and leave to soak whilst silver is washed. Wash spoons in bowl of hot soapy water, and dry whilst hot, likewise forks. Take knives out of jug, wipe with dish-cloth, and dry. Next wash tumblers and other glassware in clean warm soapy water, rinse under cold tap, and allow to drain until all are washed—they will take much less drying if done in this manner. Cups should be washed in hot soapy water (the mop comes in useful here, as it enables the washer-up to use very hot water), and when a few have been washed they should be dried while still hot. For saucers, plates, and dishes, large racks containing sufficient compartments for each size should be fitted over the sink. These articles should each be washed in turn (with the mop) in a very hot, soapy lather, rinsed separately under the cold tap as washed, and allowed to drain dry in the racks, being much cleaner and brighter than if dried with wet and dirty towels, as is frequently the case. The various tureens and other oddments should be washed and dried in the ordinary manner.

An enormous amount of breakages would be saved if not more than one article were put into the bowl at a time, especially cups, which so frequently lose their handles; but this method would be altogether too ideal for the hospital washer-up, unfortunately.

### MARCH QUESTIONS.

**(1) The Admission of Patients.**

Is it an advantage to admit patients to small hospitals (under 50 beds) by subscribers' letters, or is the system of admission on a doctor's order only preferable.

**(2) Repairs and Renewals.**

Describe the system adopted at your institution for dealing with repairs, including (a) the arrangement in force for the periodical examination of the structure, and (b) the method of collecting, recording, and disposing of repair requisitions.

### RULES.

The following rules must be observed:—

1. Contributions must be written on one side of the paper. Brevity and terseness are desirable features. The MSS. must bear the name and address of the sender and be accompanied by coupon to be cut from the back cover (inside page) of the current issue of THE HOSPITAL. A pseudonym must be chosen if the name is not to be published.

2. Contributions must be addressed to the Editor of THE HOSPITAL, Institutional Worker Supplement, 28 & 29 Southampton Street, Strand, London, W.C. 2, should reach him before the end of the current month, and be marked in the left-hand corner "Facts and Figures."

**A minimum payment of Five Shillings will be made for each published answer.**

### QUESTIONS INVITED.

IN connection with our Question Box, we offer every month five shillings each for the two best questions which are sent in for consideration. The questions may be on any institutional subject and concern any department, from the secretary's to the porter's, or the matron's to the domestic staff; the one essential is that they must be practical and deal with points that have a definite relation to hospital or institutional administration, upkeep, finance, or management. Points relating to artificers' work, housekeeping, the laundry, out-patients, and other practical matters will be welcome. It is hoped that thus, when difficult or doubtful points arise in the course of their work, institutional workers will be encouraged to put them in the form of a question and send them to the Editor, THE HOSPITAL Bureau of Information, 28 & 29 Southampton Street, Strand, W.C. 2, marked "Question Box."

The best questions will be published in due course and our readers will be given the opportunity of answering them. Thus all institutional workers may be able to co-operate with the view of helping the work and smoothing the difficulties of each other. In short, we want the Question Box of the *Institutional Worker* to be freely used by every worker habitually.

### ENQUIRIES AND ANSWERS.

### *THE SICK AND IN NEED.*

**Orphan Working School.**

THE child might possibly be admitted to the Orphan Working School and Alexandra Orphanage, Haverstock Hill, N.W., where orphan and other necessitous children are maintained and educated. Children are admitted between infancy and eleven years of age and retained until fourteen. Elections take place in May and November. Write to the Secretary of the School and Orphanage, at 73 Cheapside, London, E.C.—WORKER.

**Help Required for Lady Suffering Great Hardships.**

WE suggest that you write to the Secretary of the Universal Beneficent Society, 15 Soho Square, London, W., who will no doubt endeavour to give you the help you so urgently require. The main object of this society is to afford relief to persons who are not classed as the poor in the general acceptation of the term, but who suffer hardships and privations all the more severe because of the position they formerly held. The society either gives an annuity at the rate of £15 to infirm or afflicted persons of either sex, temporary assistance by grants of money or otherwise to deserving persons whose distress has been caused by unforeseen misfortune, or small loans on security but without interest to those needing such assistance.—DISTRESSED.

### *EMPLOYMENT AND TRAINING.*

**Board and Lodging for Part-time Services.**

WE know of no institutions in London at the present moment offering board and lodging to male students willing to give part of their time to the care of boys at clubs and hostels. We are, however, informed by the Young Men's Christian Association, 12 and 13 Russell Square, London, W.C., that it is probable that in the course of the next two or three months such positions will be offered by them in or quite near London.—H. M.

**Child Welfare Work.**

WE suggest that you write to the Secretary of the National Society of Day Nurseries, 4 Sydney Terrace, Fulham Road, London, S.W., who will possibly help you to obtain the work which you require. Or you might write to the Health Office at Kingston. We regret that we are unable to give postal replies.—B. M.

**General Training for Officer's Wife.**

HAVING regard to the facts you mention, we do not think the matrons of training-schools would be prepared in ordinary circumstances to accept you as a probationer. There are, however, exceptions to all rules, and as so much depends upon the personal equation it is possible that an application for training would meet with success, especially in view of the abnormal demand for trained nurses. We suggest that you obtain the book "How to Become a Nurse," published by The Scientific Press, Ltd., 28 and 29 Southampton Street, Strand, W.C. 2, price 2s. 10d. post free. You should also watch the advertisement columns in the nursing journals which appear each week.—L. ST. J.

**Free Massage Training.**

WE would most decidedly advise you to enter for a general training before taking up massage and electricity. You will find it possible at certain institutions to obtain this special additional training, or a part of it, free as you desire. Probationers are prepared for the examination of the Incorporated Society of Trained Masseuses at the Northampton General Hospital and at the Portsmouth Parish Infirmary. Nurses remaining for a fourth year receive massage as well as other special training. You will find particulars of other institutions in "How to Become a Nurse," published by The Scientific Press, Ltd., 28 and 29 Southampton Street, Strand, W.C. 2, price 2s. 10d. post free.—LOTTIE.

### *THE HOUSEKEEPERS' DEPARTMENT.*

**Hospital Rationing.**

As compulsory rationing of meat, butter, and margarine applies only to London and the Home Counties, your provincial institution will benefit by the experience of those hospitals situated in this area. At the time of writing it is not quite clear what limitations will be placed upon the patients and staff respectively. The rationing of military patients prescribed by the Army Council is on a fairly liberal scale, and permits of some latitude for the exercise of discretion by the officers in charge. Presumably civilian patients will be dealt with in the same way. Prior to the enforcement of the rationing scheme on February 25 hospital meat supplies had for some weeks been limited to 50 per cent of last October's supplies for the staff, the supplies for the patients remaining the same, upon production of a medical certificate. Certain hospitals have not, at the time of writing, completed the forms issued by the local Food Committees, and pending some definite instruction from the Ministry of Food respecting the rationing of staffs and patients, application should be made to the local Food Committees for emergency orders in the event of difficulty arising. It is an obligation of the managers of the hospitals to collect the patients' meat tickets when they enter the hospitals, and to cancel the coupons for the periods during which they remain in the institutions. The obligation does not, however, apply to sugar tickets.—HOSPITAL SECRETARY.

